# Geometrically modified bovine pericardium membrane promotes the expression of molecules targeted for a faster integration and vascularization process

**DOI:** 10.3389/fbioe.2024.1455215

**Published:** 2024-11-13

**Authors:** Olga Morgante, Ylenia Della Rocca, Guya Diletta Marconi, Antonella Mazzone, Marcos F. X. B. Cavalcanti, Oriana Trubiani, Francesca Diomede, Jacopo Pizzicannella

**Affiliations:** ^1^ Department of Innovative Technologies in Medicine and Dentistry, University “G. d’Annunzio” Chieti-Pescara, Chieti, Italy; ^2^ Department of Dental Clinic, Universidade Nove de Julho, SãoPaulo, Brazil; ^3^ Department of Engineering and Geology, University “G. d’Annunzio” Chieti-Pescara, Pescara, Italy

**Keywords:** tissue engineering, bovine pericardium membrane, vascular endothelial growth factor, adhesion molecules, neovascularization, human periodontal ligament stem cells

## Abstract

**Introduction:**

In recent years, advancements in technology and the refinement of engineering techniques have facilitated the development of tissue engineering, placing particular emphasis on the use of 3D-biomaterials with several structural and chemical geometric features. In particular, increasing information on biomaterial geometric surfaces has allowed for a better understanding of tissue regenerative processes. In the present study a comparison between BioRipar^®^, bovine pericardium membrane, modified with micrometric roundish regular open pores (BioR-Ps) and BioRipar^®^ without pores (BioR-NPs) has been investigated.

**Methods:**

The expression of adhesion molecules such as: fibronectin, vimentin, focal adhesion kinase (FAK), integrin 1β, integrin α5, E-cadherin, and molecules involved in neovascularization processes such as: vascular endothelial growth factor (VEGF) and vascular endothelial growth factor receptor (VEGF-R) were evaluated in an *in vitro* model containing primary culture of human periodontal ligament stem cells (hPDLSCs) through multiparametric analysis.

**Results:**

The results indicated a markedly significant expression of all the abovementioned molecules in hPDLSCs cultured withBioR-Ps compared to hPDLSCs cultured with BioR-NPs. Scanning electron microscopy analysis indicated a marked interaction between the cells and the substrate, particularly evident in the proximity of open pores in the hPDLSCs cultured on the BioR-P surface compared to hPDLSCs cultured on the BioR-NP surface. Thus, the presence of micrometric open pores on the scaffold stimulates the proliferation potential of cells apart from their adhesion ability on the patch, particularly near the pores

**Discussion:**

Expression of angiogenic molecules strengthened the performance of the modified BioR-Ps. During synthesis, 3D-biomaterial micrometric open-pores enable better bonding between cells and materials, increasing contact area and promoting cellular molecular signals in biomaterial-guided tissue engineering.

## 1 Introduction

Biomaterials respond to human needs by replacing or integrating tissues and organs damaged due to pathological or traumatic events. The human body reacts spontaneously to damage and/or injury in one of its parts through self-regenerative processes. When the tissue appears to be overly damaged, autologous tissue can be used from other regions of the patient’s body and transferred *in situ*. Biomaterials can be considered an alternative, if none of the approaches discussed are possible owing to the nature of the problem and/or its gravity.

Biomaterials used in tissue engineering for manufacturing scaffolds can be divided into two types, namely synthetic or biological, depending on their origin. The use of biological matrices is rapidly gaining interest in research and biomedical markets. Biological matrices have many advantages, including the use of complex three-dimensional matrices that naturally possess biomechanical characteristics suitable for use as implantable substitutes for the repair and regeneration of different tissues. However, drawbacks with respect to conventional scaffolds, including cell survival and integration, particularly the absence of a vascular system inside the organ, which allows nutrients, oxygen, and waste transfer are still present. Materials with different surface geometries have been proposed to increase cell adhesion, migration, and differentiation processes, as well as focal adhesion and stress fiber expression (Thery et al., 2006).

Thus, the composition and geometrical properties of the scaffolds play important roles as regulators of cell behavior; in particular, the presence of pores on the surface can represent an inductive signal contributing to the growth and organization of tissues, apart from promoting human mesenchymal stem cells (MSCs) towards the osteogenic phenotype (Fonticoli et al., 2022; Dalfino et al., 2024). Pores of different shapes may induce different tissue growth arrangements (Rumpler et al., 2008).

Biological scaffolds represent a very broad category with different characteristics and differ in the origin of the material (equine, porcine, and bovine) and the tissue from which they are made (dermis, pericardium, and submucosa). The pericardium, in particular, has shown versatility in various fields of application owing to its low rates of infection, complications, fibrotic adherence, and good hemodynamic behavior (Zouhair et al., 2020). The pericardium is composed of three layers, namely the serous layer, which is the innermost layer and comprises mesothelial cells.; the intermediate fibrous layer, which is made up of connective tissue containing oriented layers of collagen fibrils and elastin fibers; and the epipericardiumlayer, which is the outermost layer, composed of collagen bundles that form the sternopericardial ligament.

Bovine pericardium-derived membranes are widely used for repair or replacement in closure surgeries, congenital or acquired defects, and blood vessels, with low rates of infection, complications, and fibrotic adhesion, and good hemodynamic behavior in tissue engineering. It is a collagen implant material consisting of a mesh of collagen fibers after the removal of degradable proteins and decellularization (Diomede et al., 2022). They can be considered biological membranes with improved immunocompatibility and invariable biological and biomechanical properties compared to native tissues. They can be widely used for repair or replacement in closure surgeries, congenital or acquired defects, and blood vessels, with low rates of infection, complications, fibrotic adhesion, and good hemodynamic behavior.

BioRipar^®^ is a bovine pericardium collagen patch, not been chemically cross-linked, with a completely preserved structure, whose intended use is the repair of pathological or damaged tissues (Pizzicannella et al., 2019a). BioRipar^®^ has been geometrically modified with roundish micrometric open-pores (BioR-Ps). The use of perforated BioRipar^®^, optimally engineered in order to create pores of appropriate diameter at suitable distance between them, could ensure elasticity maintaining the necessary resistance and increasing cells homeostasis required in clinical practice.

Human periodontal ligament stem cells (hPDLSCs) collected from the periodontal ligament, a specialized connective tissue, representing an interface between bone socket with the tooth root surface, with multipotent and proliferative properties has been used (Winning et al., 2017), as an *in vitro* model to evaluate the performance of BioR-Ps and BioRipar^®^ (Pizzicannella et al., 2019b).

The aim of the current study was to analyze the modulation of adhesion molecules such as fibronectin, vimentin, focal adhesion kinase (FAK), integrin 1β, integrin α5, E-cadherin and molecules involved in neovascularitazion processes such as vascular endothelial growth factor (VEGF) and their receptor VEGF-R, on bovine pericardium membrane (BioRipar^®^) modified with roundish open-pores (BioR-Ps) and BioRipar^®^ without pores (BioR-NPs). Engineered BioR-Ps represent a promising material in clinical practice for many regenerative treatments, suggesting that the micrometric pores on the scaffold can be considered a structural signal that stimulates cell behavior with respect to cell proliferation potential, cell adhesion ability, and neovascularization, as evidenced by the marked expression of angiogenic markers.

## 2 Materials and methods

### 2.1 Pore creation in No-cross-linked BioRipar^®^ membranes

No-Cross-Linked BioRipar^®^ device were derived from bovine pericardium after a multiphasic decellularization, owned by Assut Europe SpA. Starting from a 5 cm × 8.56 cm (dry type with code AEPB050-086S and lot 19NG0005), sections of size 1 × 1 cm were obtained by cutting the original membrane under a laminar flow hood. The small membranes were divided into two groups, namely BioRipar^®^ with No Pores (BioR-NPs) and BioRipar^®^ with pores (BioR-Ps). The holes in the BioR-Ps were created using punch pliers with a diameter of approximately 0.1 cm and a distance of at least 0.1 cm. Holes were drilled to allow effective drainage of fluids and facilitate the passage of substances in and out of the patch. They were homogeneously present in the membranes, with four pores in each portion.

### 2.2 Ethics approval and consent to participate

The study protocol of the present paper was approved by the Ethics Committee of “G. d’Annunzio,” Chieti, Italy (prot. n. 266/17). The Department of Innovative Technologies in Medicine and Dentistry and the Laboratory of Stem Cells and Regenerative Medicine were certified according to the quality standard ISO 9001:2008 (certificate no. 32031/15/S). All patients recruited for this study completed and signed an informed consent form.

### 2.3 Cell cultures

Cell cultures were set up with hPDLSCs isolated from five human premolar teeth extracted for orthodontic reasons from different patients who provided informed consent. Specifically, the collected small tissue fragments biopsies were obtained, using a Gracey’s curette, from the third coronal area of periodontal ligament of patients with healthy general condition and without oral or systemic diseases. Tissue fragments were placed in a Petri dish with TheraPEAK™MSCGM™ Mesenchymal Stem Cell Growth Medium BulletKit™ (Lonza, Swizzerland) containing the serum free media TheraPEAK™MSCGM™ Mesenchymal Stem Cell Growth Medium and TheraPEAK™MSCGM™ Supplement. The media was replaced with fresh medium every 2 days. After 3–4 weeks, hPDLSCs that spontaneously migrated from tissue fragments were subcultured at passage 4 and were used for subsequent experiments.

### 2.4 Cell characterization

Human PDLSCs phenotype was performed using flow cytometry. Passage 2 of hPDLSCs were detached with 0.1% EDTA trypsin (Lonza) and resuspended in 1 mL PBS (Lonza). The cells were then incubated with anti-CD14-FITC (Miltenyi Biotec, Bergisch Gladbach, Germany); OCT3/4-PE, CD73-PE, SOX2-Alexa488, CD90-FITC (Becton Dickinson, San Jose, CA, United States), and CD34-PE (Beckman Coulter, Fullerton, CA, United States). After incubation with appropriate secondary antibodies, fixation in 1 mL PBS 0.5% paraformaldehyde and washing, cells were analyzed using a FACStar-plus flow cytometry system and the FlowJo™ software v10.0.7 (TreeStar, Ashland, OR, United States).

To evaluate the mesengenic differentiation hPDLSCs were iduced to adipogenic and osteogenic lineage. Cells were incubated in MSCGM-CD (Lonza) medium with osteogenic supplements and adipogenesis induction/maintenance medium (Lonza), for 21 and 28 days respectively. Both the mesengenic differentiation were confirmed by means of colorimetric assay as previously described by Rajan et al. (2017).

### 2.5 Experimental design

The experimental design is reported as follows:

1. hPDLSCs cultured on BioRipar^®^ membranes with No-Pores (BioR-NPs)
2. hPDLSCs cultured on BioRipar^®^ with Pores (BioR-Ps);

All the experiments were performed in triplicate.

### 2.6 Optical microscope

After 1 week, the morphology of the hPDLSCs cultured on BioR-NPs and BioR-Ps was observed using an inverted light microscope (Leica DMIL, Leica Microsystem).

### 2.7 Analysis using the scanning electron microscopy (SEM)

The hPDLSCs were seeded onto BioR-NPs and BioR-Ps for 1 week, fixed for 1 h in 2.5% glutaraldehyde, dehydrated using an ethanol series (35%, 50%, 70%, 80%, 90%, 95%, and 100%), and chemically dried with hexamethyldisilazane (HMDS). The samples were then mounted on aluminum stubs with double-sided carbon tape and metallized in Au by sputtering (AGAR autosputter coater) before imaging using SEM (Thermo Fisher Apreo 2 LoVac).

### 2.8 Immunofluorescence analysis

The hPDLSCs were cultured in a chamber slide with 8-wells (Ibidi, Gräfelfing, Germany) at a concentration of 6.400 cells/well. After 1 week, the cells were fixed with 4% paraformaldehyde for 1 h at room temperature. The samples were permeabilized with 0.1% Triton X-100 for 6 min and saturated with 5% skim milk in PBS for 1 h at room temperature. The primary antibodies fibronectin (sc-8422, Santa Cruz Biotechnologies, Dallas, TX, United States), vimentin (sc-6260, Santa Cruz Biotechnologies), E-cadherin (sc-8426 Santa Cruz Biotechnologies), FAK (sc- 271126 Santa Cruz Biotechnologies), integrin β1 (sc 374429 Santa Cruz Biotechnologies), integrin α5 (sc-376199 Santa Cruz Biotechnologies), VEGF (sc-7269 Santa Cruz Biotechnologies), and Flt-1 (sc-271789 Santa Cruz Biotechnologies) were incubated (concentration 1:200) overnight at 4°C. The secondary antibody Alexa Fluor 568 red fluorescence conjugated goat anti-mouse (A11031, Invitrogen, Eugene, OR, United States) was incubated (concentration 1:200) for 1 h at 37°C and successively cytoskeleton and nuclei were stained respectively with 1:200 Alexa Fluor 488 phalloidin green fluorescence conjugate (A12379, Invitrogen) and 1:200 TOPRO (T3605, Invitrogen) for 1 h at 37°C. Samples were observed using a Zeiss LSM800 META confocal microscope (Zeiss, Jena, Germany) connected to an inverted Zeiss Axiovert 200 microscope equipped with a Plan Neofluar oil immersion objective (×40/1.3 NA). Images were collected using an argon laser beam with excitation lines at 488 nm and a helium-neon source at 543 and 633 nm. Localization analysis was performed offline on images acquired using Zeiss ZEN software (ver. 2.3), positive cells were quantified based on the images collected randomly for each samples through Image J software (Diomede et al., 2020b).

### 2.9 Western blot analysis

Protein expression of the hPDLSCs was evaluated using western blot analysis as follows, briefly, the cell lysate was recovered by scraping cells cultured in 6 MW at a concentration of 80.000 cells/well and lysing using RIPA lysis and extraction buffer (Thermo Fisher Scientific, Milan, Italy). The lysate was then centrifugated at 16,000 g for 15 min at 4°C to eliminate the debris and it was quantized using a BioSpectometer (Eppendorf, Hamburg, Germany). Approximately, 50 µg ptotein was used to perform sodium dodecyl-sulfate polyacrylamide gel electrophoresis (SDS-PAGE) and western blot analysis (Bio-Rad V3 Western Workflow™, Milan, Italy). After protein transfer onto PVDF membranes (Immobilon-P transfer membrane; Millipore), the membranes were saturated with 5% skim milk in PBS for 2 h at room temperature. Post which, the membranes were incubated at 4°C overnight with the primary antibodies fibronectin (1:500) (sc-8422, Santa Cruz Biotechnologies), vimentin (1:500) (sc-6260, Santa Cruz Biotechnologies), E-cadherin (1:500) (sc-8426 Santa Cruz Biotechnologies), FAK (1:500) (sc- 271126 Santa Cruz Biotechnologies), Integrin β1 (1:500) (sc 374429 Santa Cruz Biotechnologies), Integrin α5 (1:500) (sc-376199 Santa Cruz Biotechnologies), VEGF (1:500) (sc-7269 Santa Cruz Biotechnologies), and Flt-1 (1:500) (sc-271789 Santa Cruz Biotechnologies). The day after, the secondary antibody peroxidase-conjugated anti-mouse (1:5,000) (A90-116P, Bethyl Laboratories Inc., Montgomery, Texas, United States) was added to it and was incubated for 1 h at room temperature and the membranes were read with an Alliance 2.7 system (Uvitec Ltd., Cambridge, United Kingdom) to identify and quantify the protein bands (Trubiani et al., 2012). The data were normalized with the protein expression of β-Actin (1:750) (sc-47778, Santa Cruz Biotechnology) used as loading control. All the experiments were performed in triplicate.

### 2.10 Real-time-PCR analysis

Total RNA was extracted from the cell lysate recovered in a 6 multiwell (MW) with 80.000 cells/well. RNA was isolated using the PureLink RNA Mini Kit (Ambion, Thermo Fisher Scientific, Milan, Italy) and quantified at 260 nm using NanoDrop 2000 ultraviolet-visible (UV-Vis) spectrophotometer (Thermo Fisher Scientific, Waltham, MA, United States). Approximately, 1 μg RNA was reverse transcribed to cDNA according to the M-MLV Reverse Transcriptase (M1302 Sigma-Aldrich,) protocol. Evaluation of fibronectin (Hs.PT.58.40005963, Tema Ricerca Srl), vimentin (Hs.PT.58.38906895, Tema Ricerca Srl), CDH1 (Hs.PT.58.3324071, Tema Ricerca Srl), PTK2 (Hs.PT.58.524947, Tema Ricerca Srl), ITGB1 (Hs.PT.58.39883300, Tema Ricerca Srl), ITGA5 (Hs.PT.58.4796384, Tema Ricerca Srl), VEGFA (Hs.PT.58.21234833, Tema Ricerca Srl), and FLT1 (Hs.PT.58.40906831, Tema Ricerca Srl) gene expression was performed using the Mastercycler ep realplex real-time PCR system (Eppendorf) and the PrimeTime Gene Expression Master Mix (Tema Ricerca Srl). The amplification program included a preincubation step for cDNA denaturation (3 min at 95°C), followed by 40 cycles consisting of a denaturation step (15 s at 95°C) and an annealing step (1 min at 60°C). Expression levels for each gene were obtained according to the 2^−ΔΔCT^ method considering B2M (Tema Ricerca Srl) as a housekeeping gene. RT-PCR was performed in two independent experiments and triplicate determinations were performed for each sample.

### 2.11 ELISA assay

The supernatants collected from hPDLSCS + BioR-NPs and hPDLSCs + BioR-Ps cultures, were analyzed for VEGF level through ELISA assay performed following the protocol described in Human VEGF ELISA Kit (Invitrogen, Thermo Fisher, KHG0111).

### 2.12 Statistical analysis

Statistical evaluation was performed using GraphPad Prism 4.0 software (Graph-Pad, San Diego, CA, United States) using the *t*-test and ordinary one-way ANOVA, followed by *post hoc* Bonferroni’s multiple comparison tests. The ‘fold change’ of gene expression levels was calculated with the 2^−ΔΔCT^ method. Statistical significance was set at *p* < 0.01. GraphPad Prism 10.0 software was used to determine the standard curve for the ELISA and to calculate VEGF concentration in pg/mL.

## 3 Results

### 3.1 Cytofluorimetric analysis and mesengenic differentiation of hPDLSCs

In order to confirm hPDLSCs phenotype, flow cytometry analysis was conducted on stem cells at the second passage. *Ex vivo* expanded hPDLSCs showed positivity for Oct3/4, Sox-2, CD73, and CD90. On the contrary, the hematopoietic stem cell markers as CD14, and CD34 were negative (Supplementary Figure S1A).

Light microscopy investigations showed plastic adherent hPDLSCs with spindle shaped morphology with elongated cytoplasmic processes (Supplementary Figure S1B). To evaluate the adipogenic differentiation abilty of hPDLSCs, the cellular monolayer was stained with Oil Red O and observed by light microscopy. Adipogenic-induced cells showed the fine evident intracellular lipid droplets at cytoplasmic level (Supplementary Figure S1D). Alizarin Red S stained confluent cells showed after 21 days of osteogenic induction a characteristic positive staining for calcium deposition (Supplementary Figure S1C).

### 3.2 The hPDLSCs morphological analysis

**FIGURE 1:**
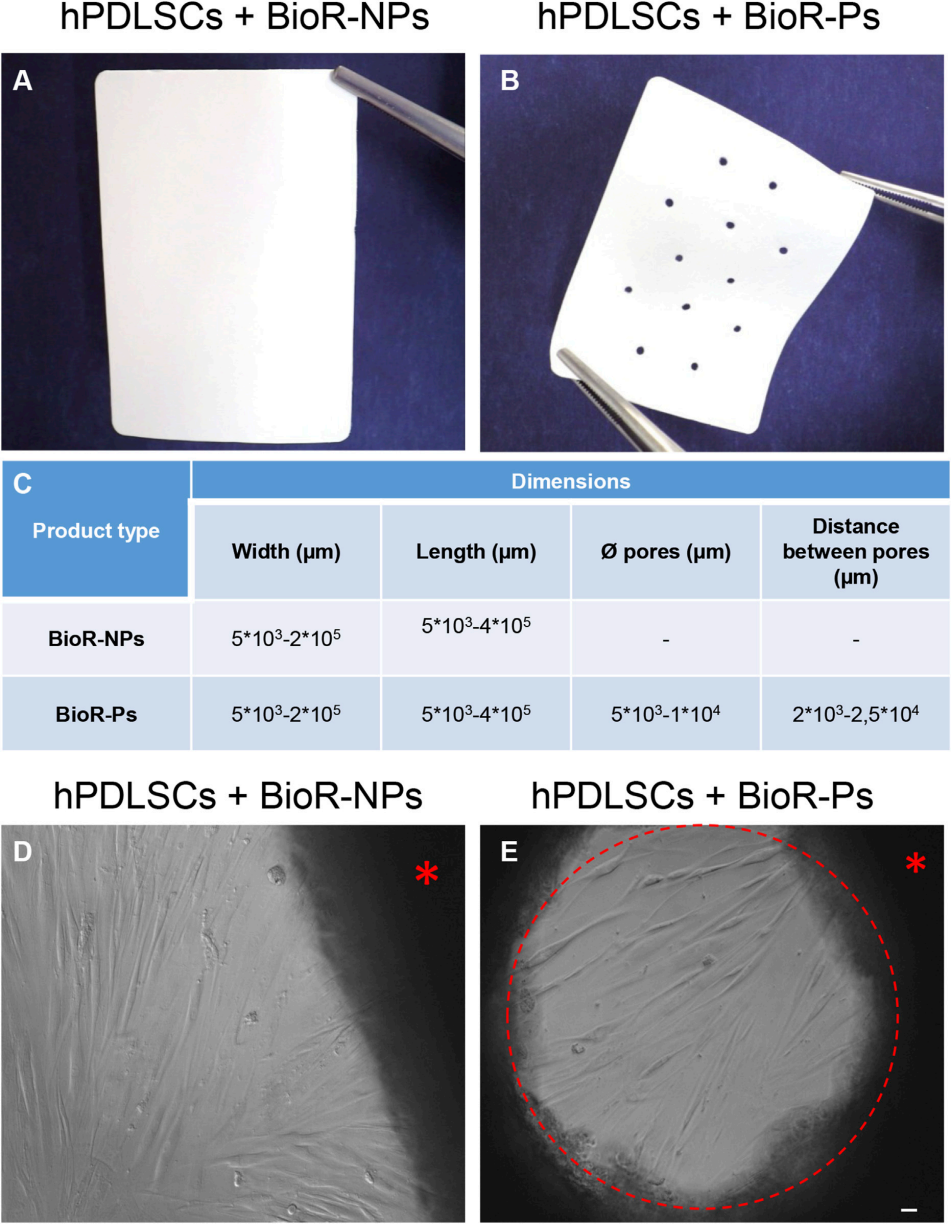
Photographs of **(A)** BioRipar^®^ (BioR)-NPs and **(B)** BioR-Ps. **(C)** Table of features of BioR-NPs and BioR-Ps. **(D, E)** The morphology of hPDLSCs + BioR-NPs and hPDLSCs + BioR-Ps was observed at the inverted light microscope. Red asterisk: BioR membrane. Red circle: pore of the BioR-Ps. Scale bar 20 µm.

**FIGURE 2:**
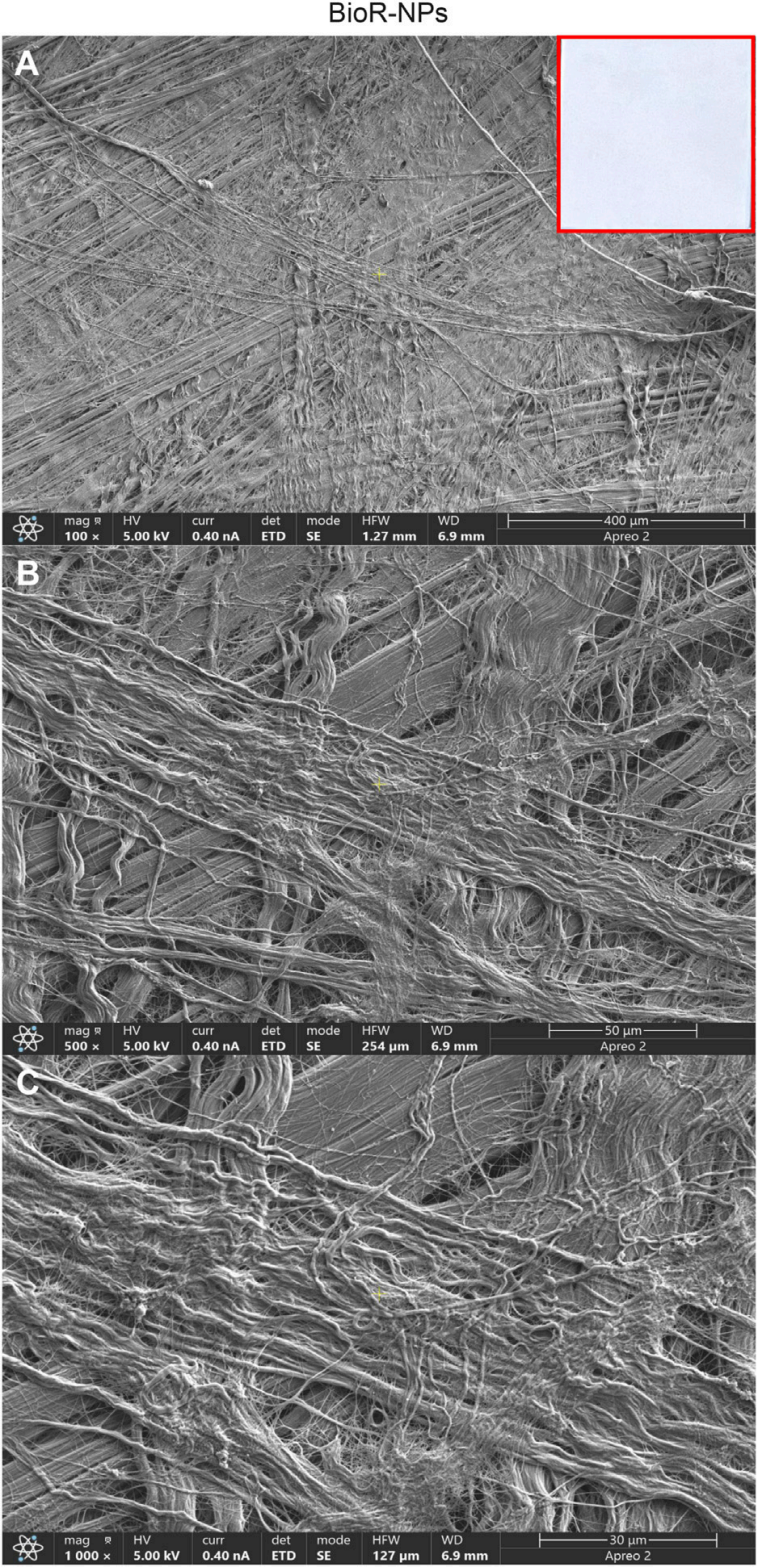
Representative images showing at different magnification the features of BioR-NPs membrane by means scanning electron microscopy. Cross-banded and parallel fibrils are mainly viewable. BioRipar®-NPs at **(A)** Mag: 100X. **(B)** Mag: 500X. **(C)** Mag: 1,000X. Insert in red square: BioRipar®-NPs membrane.

**FIGURE 3:**
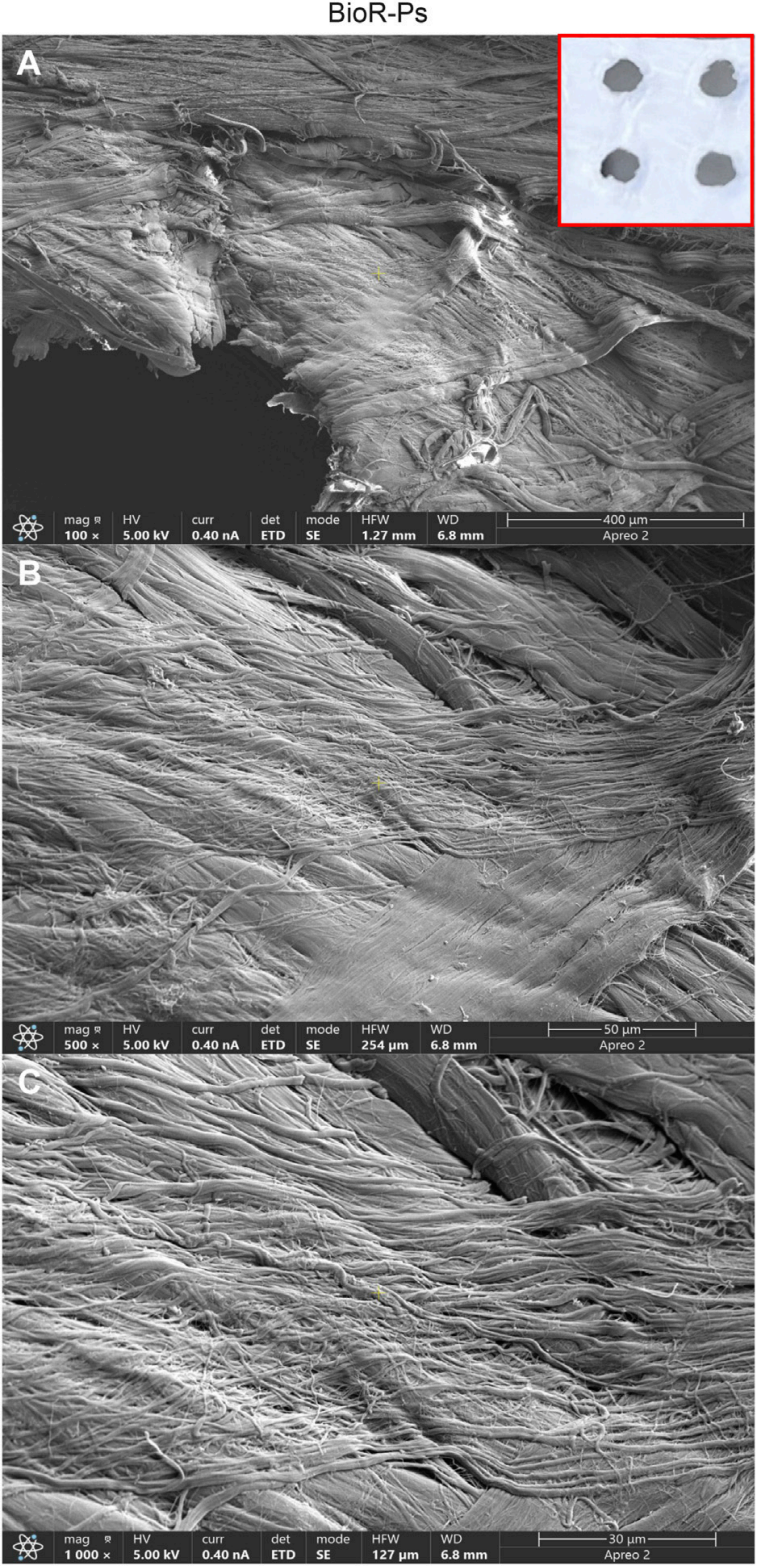
Representative images showing at different magnification the features of Bio-Ps membrane by means scanning electron microscopy. Cross-banded and parallel fibrils are mainly viewable. The presence of holes approximately at 0.1 cm are evident. BioR-Ps at **(A)** Mag: 100X. **(B)** Mag: 500X. **(C)** Mag: 1,000X. Insert in red square: BioR-Ps membrane.

**FIGURE 4:**
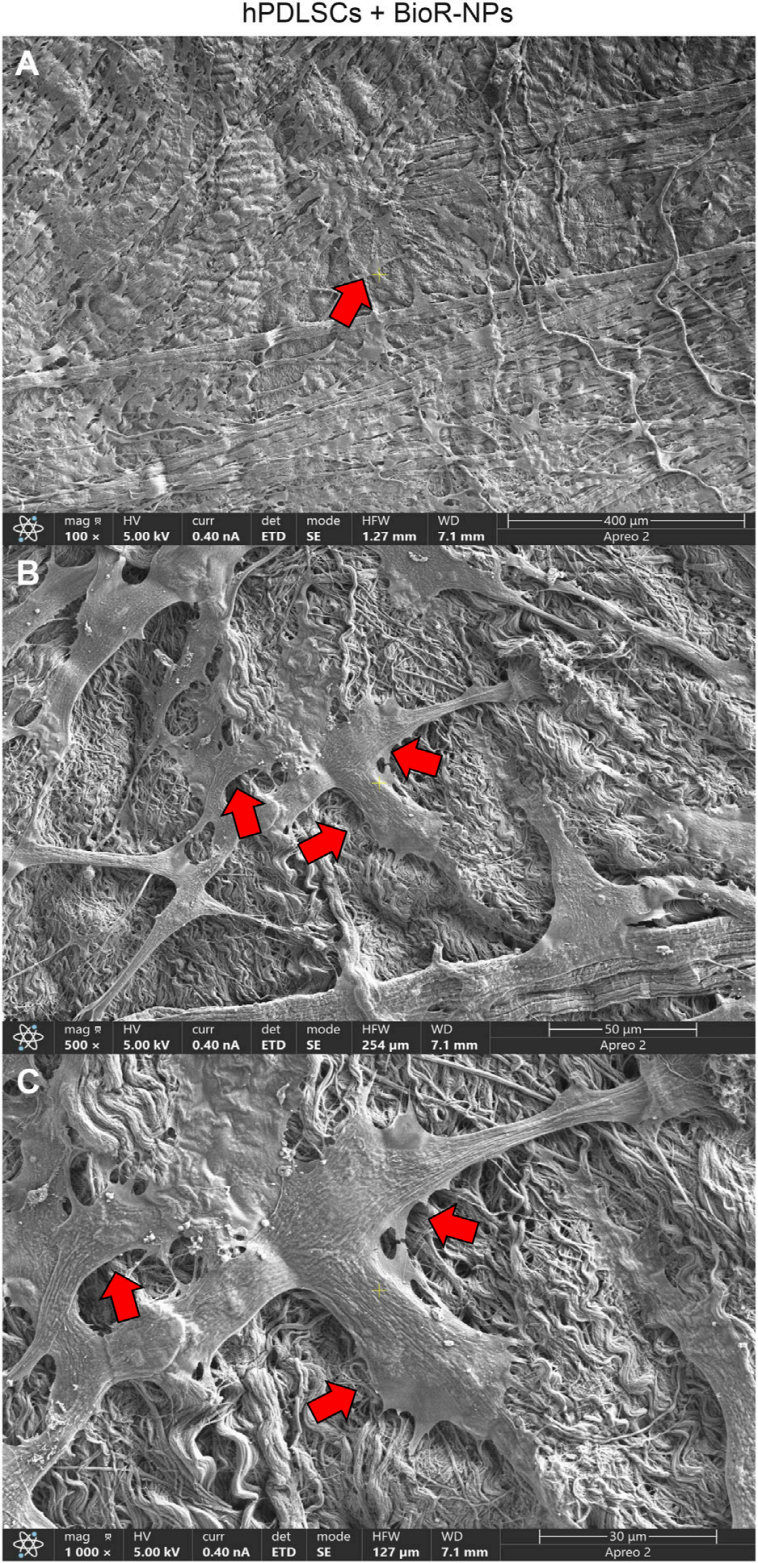
**(A–C)** Representative images of primary cultures of hPDLSCs after 1 week of culture seeded on BioR-NPs. hPDLSCs with fibroblast-like shape cultured on the BioR-NPs (red arrows). Contact zones between confluent cells are visible. Higher magnification images showed the cells that make contact and cover the biomaterial geometry surface. In particular marked interactions between the cells and the substrate is obtained through extending cytoplasmic elongation and filopodia more evident at pore level, indiscernible for the intimate contact between neighbouring cells. At higher magnification, a strongly adhesion of hPDLSCs on substrate is appreciable. BioR-NPs at **(A)** Mag: 100X. **(B)** Mag: 500X. **(C)** Mag: 1,000X.

**FIGURE 5:**
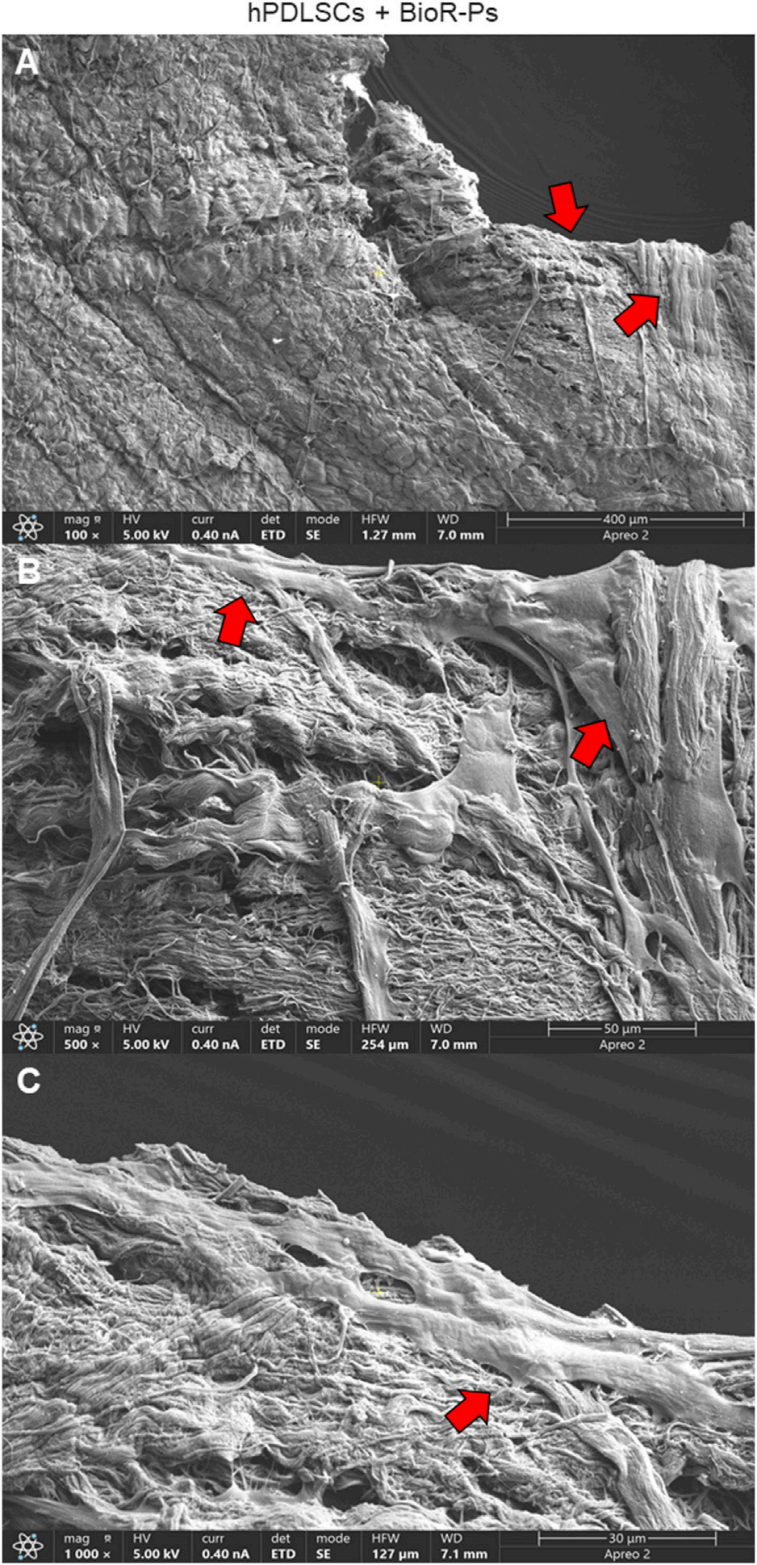
Microphotograph of a primary culture of hPDLSCs seeded on BioR-NPs. **(A)** Living cells adhered on substrate showing a morphological homogeneous fibroblast-like appearance with a stellate shape and elongated cytoplasmic processes. **(B, C)** Cells colonized the biomaterial and take direct junctional-like contact with each other (red arrows). BioR-NPs at **(A)** Mag: 100X. **(B)** Mag: 500X. **(C)** Mag: 1,000X.

After 1 week of culture, the morphology of hPDLSCs cultured on BioR-NPs and BioR-Ps was observed under an inverted light microscope. No morphological differences were observed among the experimental conditions under an inverted light microscope (Figures 1D, E). Human PDLSCs were observed under a scanning electron microscope at high magnification to evaluate their morphological features. BioR-NPs and BioR were observed under SEM to analyze their surfaces without cell culture (Figures 2, 3), and those cultured for 1 week on BioR-NPs showed a fibroblast-like shape (Figure 4). Contact zones between confluent cells were also visible. Images that were highly magnified showed the cells that made contact with and covered the biomaterial surface. In particular, marked interactions between the cells and the substrate are obtained by extending cytoplasmic elongation, and filopodia are more evident at the pore level, which is indiscernible for the intimate contact between neighboring cells. At higher magnification, strong adhesion of hPDLSCs to the substrate was observed (Figure 4C). Cells cultured on the BioR-Ps showed good surface adhesion and a morphologically homogeneous fibroblast-like appearance with a stellate shape and elongated cytoplasmic processes. The cells colonized the biomaterial and formed direct junctional-like contacts with each other (Figure 5).

### 3.3 Protein expression observed through CLSM and western blot analyses

**FIGURE 6:**
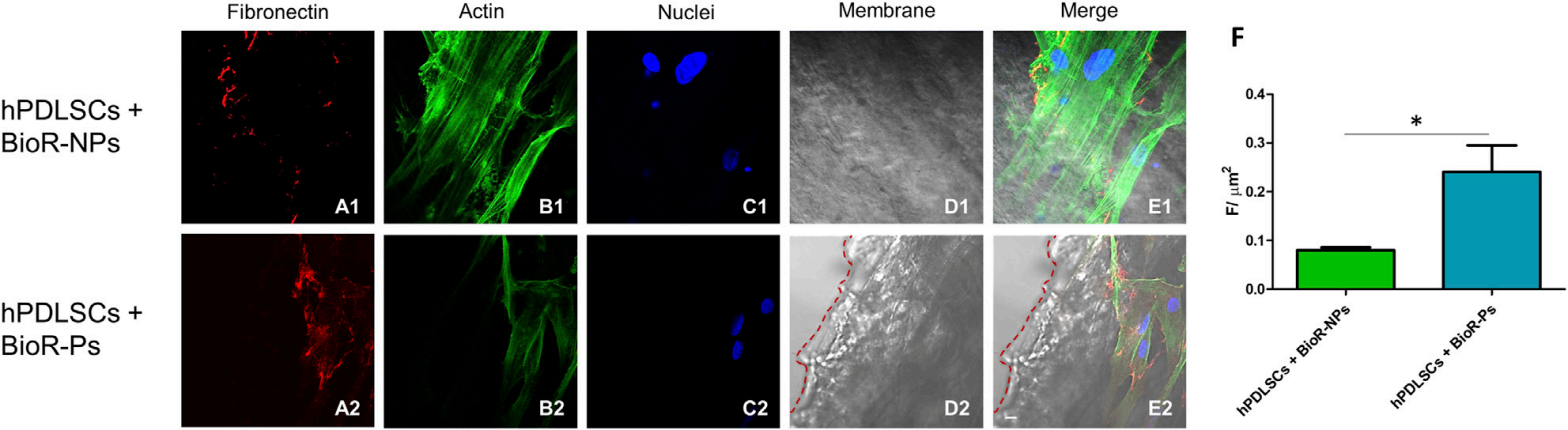
Fibronectin expression analyzed by confocal microscopy. Expression of Fibronectin, **(A1–E2)** was evaluated in hPDLSCs + BioR-NPs and hPDLSCs + BioR-Ps after 1 week of culture. Red fluorescence: Fibronectin **(A1–A2)**; Green fluorescence: cytoskeleton actin **(B1–B2)**; Blue fluorescence: cell nuclei **(C1–C2)**, BioR membranes **(D1–D2)**; Merge **(E1–E2)**. **(F)** Histograms represent the positive cells for the analyzed marker. *p < 0.05. Scale bar: 20 μm.

**FIGURE 7:**
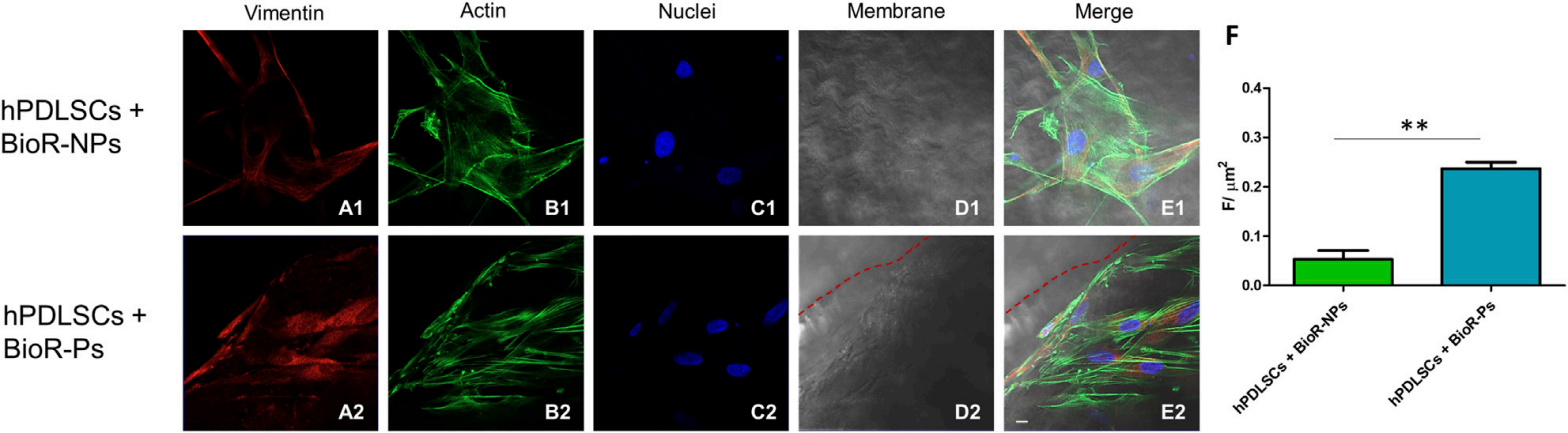
Vimentin expression analyzed by confocal microscopy. Expression of Vimentin **(A1–E2)** was evaluated in hPDLSCs + BioR-NPs and hPDLSCs + BioR-Ps after 1 week of culture. Red fluorescence: Vimentin **(A1–A2)**; Green fluorescence: cytoskeleton actin **(B1–B2)**; Blue fluorescence: cell nuclei **(C1–C2)**, BioR membranes **(D1–D2)**; Merge **(E1–E2)**. **(F)** Histograms represent the positive cells for the analyzed marker. **p < 0.01. Scale bar: 20 μm.

**FIGURE 8:**
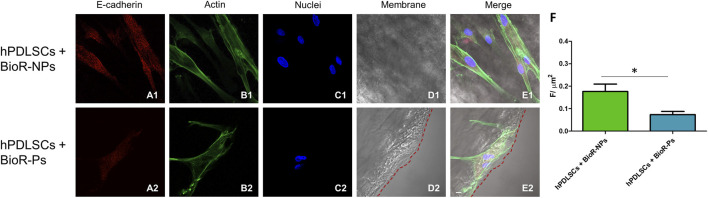
E-cadherin expression analyzed by confocal microscopy. Expression of E-cadherin **(A1–E2)** was evaluated in hPDLSCs + BioR-NPs and hPDLSCs + BioR-Ps after 1 week of culture. Red fluorescence: E-cadherin **(A1–A2)**; Green fluorescence: cytoskeleton actin **(B1–B2)**; Blue fluorescence: cell nuclei **(C1–C2)**, BioR membranes **(D1–D2)**; Merge **(E1–E2)**. **(F)** Histograms represent the positive cells for the analyzed marker. *p < 0.05. Scale bar: 20 μm.

**FIGURE 9:**
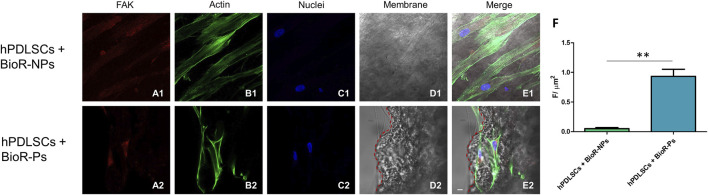
FAK expression analyzed by confocal microscopy. Expression of FAK **(A1–E2)** was evaluated in hPDLSCs + BioR-NPs and hPDLSCs + BioR-Ps after 1 week of culture. Red fluorescence: FAK **(A1–A2)**; Green fluorescence: cytoskeleton actin **(B1–B2)**; Blue fluorescence: cell nuclei **(C1–C2)**, BioR membranes **(D1–D2)**; Merge **(E1–E2)**. **(F)** Histograms represent the positive cells for the analyzed marker.**p < 0.01. Scale bar: 20 μm.

**FIGURE 10:**
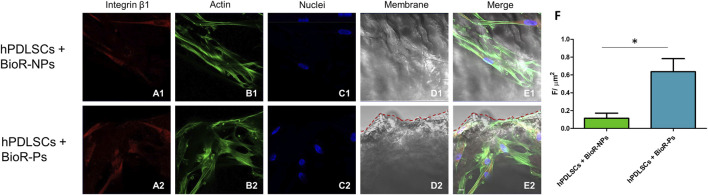
Integrin β1 expression analyzed by confocal microscopy. Expression of Integrin β1 **(A1–E2)** was evaluated in hPDLSCs + BioRipar®-NPs and hPDLSCs + BioRipar®-Ps after 1 week of culture. Red fluorescence: Integrin β1 **(A1–A2)**; Green fluorescence: cytoskeleton actin **(B1–B2)**; Blue fluorescence: cell nuclei **(C1–C2)**, BioRipar^®^ membranes **(D1–D2)**; Merge **(E1–E2)**. **(F)** Histograms represent the positive cells for the analyzed marker. *p < 0.05. Scale bar: 20 μm.

**FIGURE 11:**
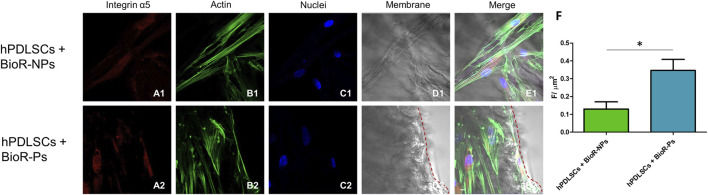
Integrin α5 expression analyzed by confocal microscopy. Expression of Integrin α5 **(A1–E2)** was evaluated in hPDLSCs + BioR-NPs and hPDLSCs + BioR-Ps after 1 week of culture. Red fluorescence: Integrin α5 **(A1–A2)**; Green fluorescence: cytoskeleton actin **(B1-B2)**; Blue fluorescence: cell nuclei **(C1–C2)**, BioR membranes **(D1–D2)**; Merge **(E1–E2)**. **(F)** Histograms represent the positive cells for the analyzed marker. *p < 0.05. Scale bar: 20 μm.

**FIGURE 12:**
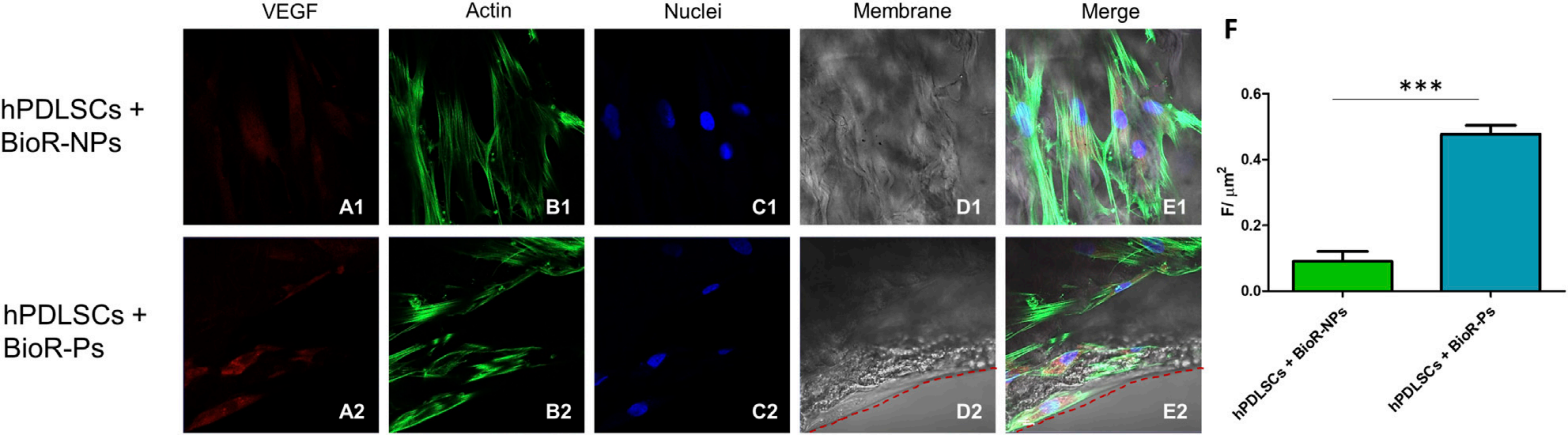
VEGF expression analyzed by confocal microscopy. Expression of VEGF **(A1–E2)** was evaluated in hPDLSCs + BioR-NPs and hPDLSCs + BioR-Ps after 1 week of culture. Red fluorescence: VEGF **(A1–A2)**; Green fluorescence: cytoskeleton actin **(B1–B2)**; Blue fluorescence: cell nuclei **(C1–C2)**, BioR membranes **(D1–D2)**; Merge **(E1–E2)**. **(F)** Histograms represent the positive cells for the analyzed marker.***p < 0.001. Scale bar: 20 μm.

**FIGURE 13:**
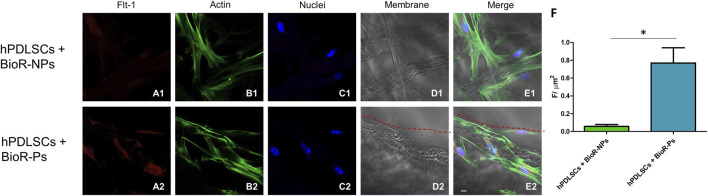
Flt-1 expression analyzed by confocal microscopy. Expression of Flt-1 **(A1–E2)** was evaluated in hPDLSCs + BioR-NPs and hPDLSCs + BioR-Ps after 1 week of culture. Red fluorescence: Flt-1 **(A1–A2)**; Green fluorescence: cytoskeleton actin **(B1–B2)**; Blue fluorescence: cell nuclei **(C1–C2)**, BioR membranes **(D1–D2)**; Merge **(E1–E2)**. **(F)** Histograms represent the positive cells for the analyzed marker. *p < 0.05. Scale bar: 20 μm.

**FIGURE 14:**
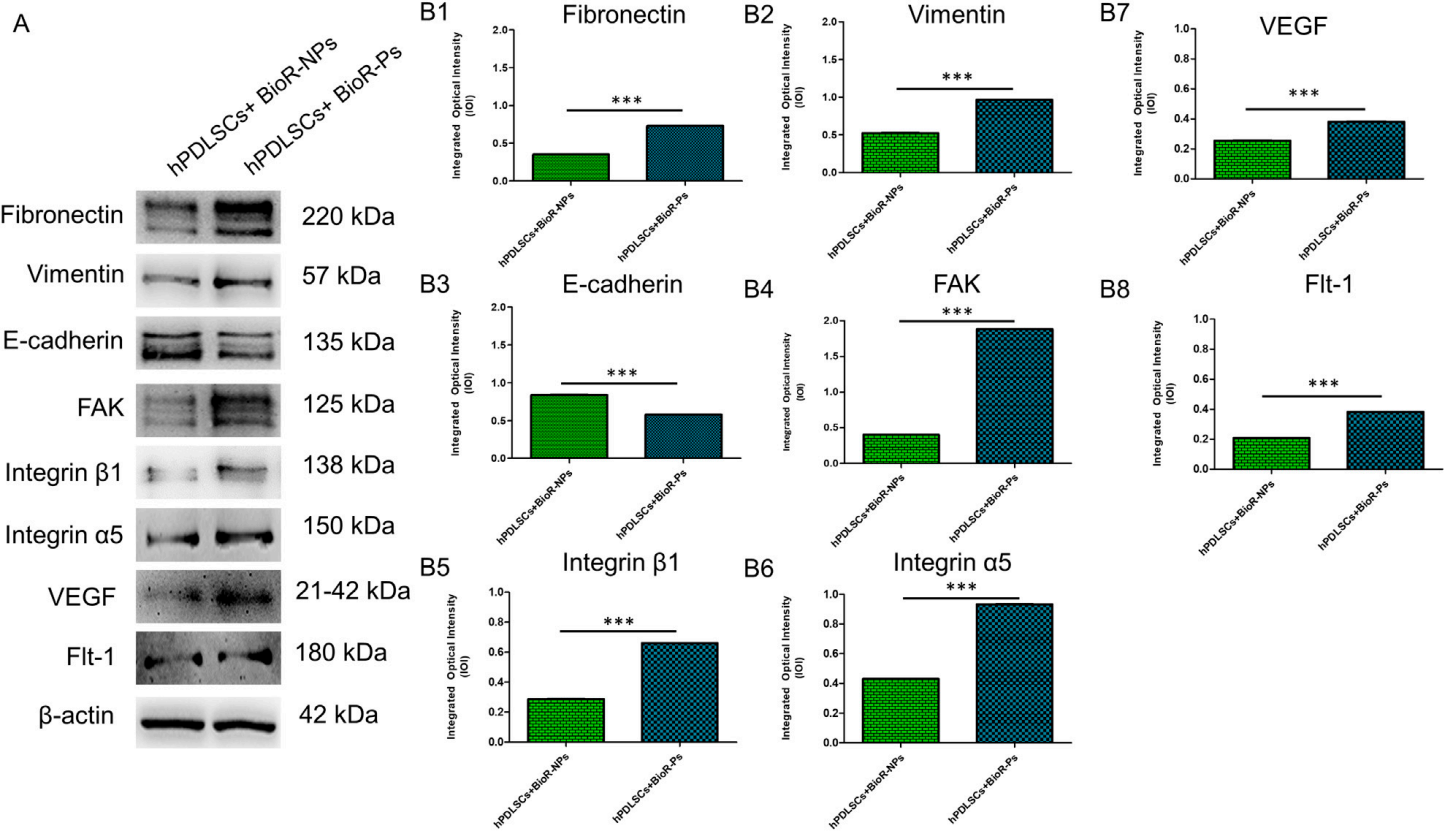
Western blotting analysis. Fibronectin, Vimentin, E-cadherin, FAK, Integrin β1, Integrin α5, VEGF and Flt-1 protein expression in hPDLSCs + BioR-NPs and hPDLSCs + BioR-Ps after 1 week of culture **(A)** Each membrane was probed with β-actin antibody to verify the loading consistency. **(B1–B8)** Histograms represent densitometric measurements of protein bands expressed as the integrated optical intensity (IOI) mean of three separate experiments. The error bars show the standard deviation (±SD). *p < 0.05; **p < 0.01; ***p < 0.001.

The immunofluorescence images showed the expression of fibronectin, vimentin, E-cadherin, FAK, integrin β1, integrin α5, VEGF, and Flt-1 in hPDLSCs cultured on BioR-NPs and BioR-Ps for 1 week. The results showed that fibronectin, vimentin, FAK, integrin β1, integrin α5, VEGF, and Flt-1 were significantly expressed in hPDLSCs + BioR-Ps compared to hPDLSCs + BioR-NPs, at the same time, E-cadherin was significantly expressed in hPDLSCs + BioR-Ps than in hPDLSCs + BioRipar®-NPs (Figures 6–13). The results obtained by western blot analysis confirmed the immunofluorescence data (Figure 14).

### 3.4 Genes expression

**FIGURE 15:**
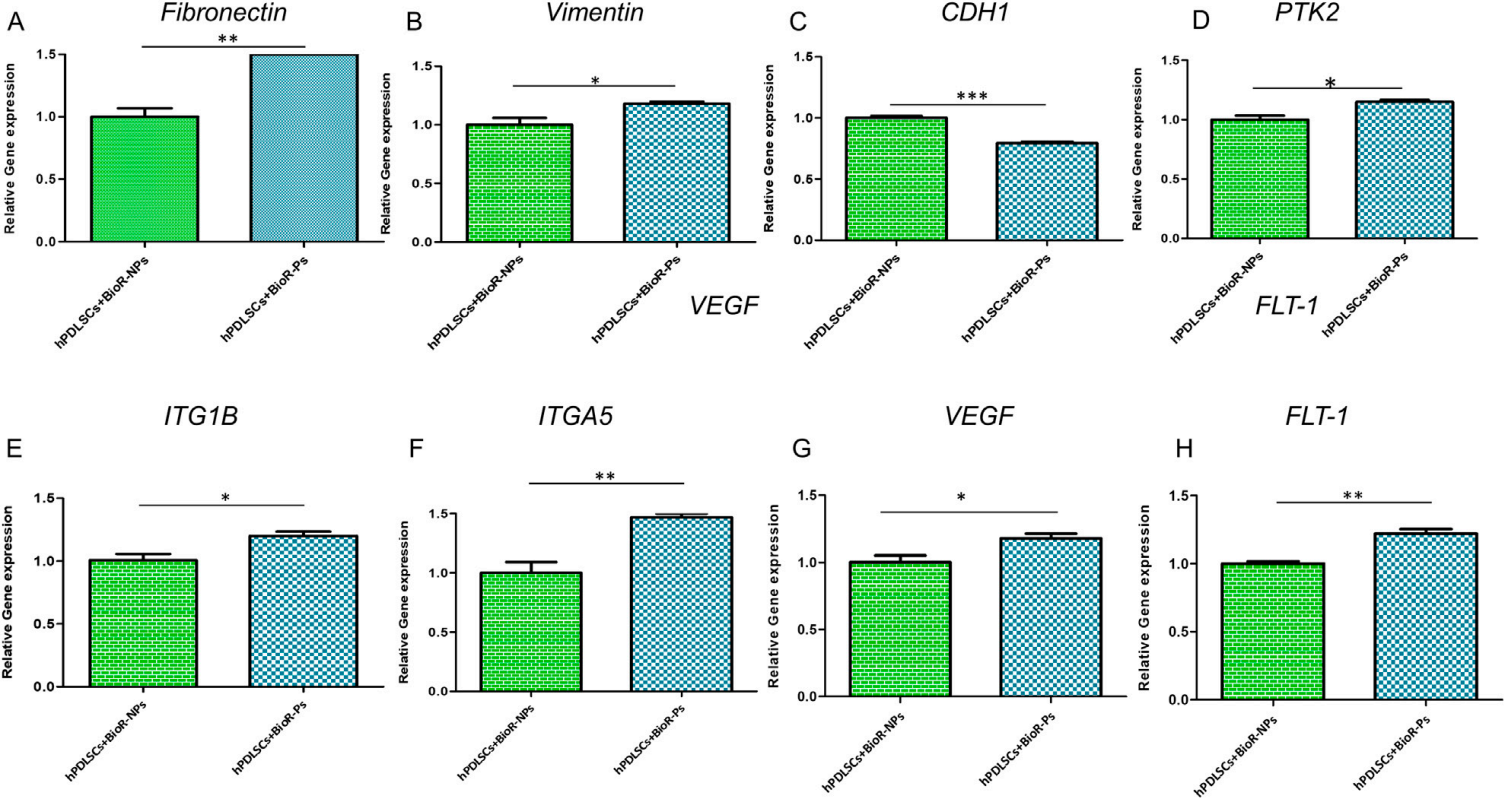
**(A–H)** Histograms of RT-PCR showed the mRNA levels of Fibronectin, Vimentin, CDH1, PTK2, ITG1B, ITGA5, VEGF and FLT-1 in hPDLSCs + BioR-NPs and hPDLSCs + BioR-Ps after 1 week of culture *p < 0.05; **p < 0.01; ***p < 0.001.

The histogram shows the gene expression of fibronectin, vimentin, CDH1, PTK2, ITG1B, ITGA5, VEGF, and FLT1 in hPDLSCs + BioR-NPs and hPDLSCs + BioR-Ps after 1 week of culturing (Figure 15). The results obtained by real-time PCR confirmed the qualitative results obtained by CLSM and western blot analyses.

### 3.5 VEGF level

The data obtained from the ELISA assay analysis confirmed the increase in VEGF levels in hPDLSCs + BioR-Ps compared to hPDLSCs + BioR-NPs at the supernatant level.

## 4 Discussion

Ideally, a medical device should provide biological, chemical, and mechanical properties corresponding to those of the tissue to be replaced to favor tissue regeneration. Biological materials respond better to these properties than synthetic materials, and the structural and mechanical characteristics of engineered biomaterials are fundamental to the design and optimization of biological implants (Badylak and Gilbert, 2008). The main purpose of using biological materials is not limited only to tissue repair, but also to tissue regeneration. Implanted biomaterials promote the formation of new blood vessels and neotissue deposition by stimulating the synthesis of new extracellular matrix (ECM) elements (Bellows et al., 2007). The active degradation/substitution process of the implanted acellular tissue and tissue regeneration depends on the degree of cross-linking of the biomaterial structure. Therefore, the choice of tissue to be used also plays an important role in tissue engineering (Liang et al., 2004).

The pericardium membrane, a biological biomaterial derived from different tissue sources through the process of decellularization, which ensures the removal of cellular components while preserving ECM composition, has attracted considerable interest in regenerative medicine and tissue engineering applications (Black, 1988; Abdulghani and Mitchell, 2019).

BioRipar^®^ membrane was developed through a multiphasic process from the pericardium (DOI 10.4081/ejh.2019.3064). The decellularization process promotes good preservation of the structure of the ECM and it also preserves the mechanical properties of the native pericardium, supporting the use of the BioRipar^®^ in regenerative surgery (Bielli et al., 2019). BioRipar^®^’s properties appear to be better than competing biomeshes, as demonstrated by various studies in literature (Bielli et al., 2019; Bernardini et al., 2020). With respect to the mechanical properties, Bioripar^®^ membranes were able to hold statistically high tensile stress (Bielli et al., 2019). In the breast reconstruction carried out on an animal model, BioRipar^®^’s performance turned out to be better than other commercial biomaterials that were associated with a higher inflammation rate (Bernardini et al., 2020). BioRipar^®^ can be used for soft tissue repair, for muscle flaps reinforcement, for tendon structures reinforcement, augmentation and covering, for reinforcement and/or replacement of connective tissue, to prevent the formation of adhesions and accelerate tissue recovery time in: abdominal, cardiac and thoracic surgery, urology, gynecology, vascular surgery, orthopaedics, plastic surgery, andrology, oral surgery, traumatology, implantology, and periodontology (Horn et al., 2023). Based on the implantation site and user request, it can be manufactured in different shapes and sizes, as wet or dry, and with or without holes. Perforated membranes provide greater permeability to liquids and are preferred in all applications in which this feature is available. The holes could affect the process of tissue regeneration, as they are intended to encourage the infiltration of prosthetic tissue into specific areas on its surface and allow effective drainage of hematomas and seromas.

In the present study, in order to evaluate the cell growth and adhesion capacity of hDPLSCs on the two different surfaces, BioR-Ps and BioR-NPs in an *in vitro* model of hDPLSCs a comparison between BioRipar^®^ with pores (BioR-Ps) and BioRipar^®^ without pores (BioR-NPs) was performed In the expression of adhesion molecules was evaluated by qualitative and quantitative analyses using immunofluorescence, western blotting, and real-time PCR. Adhesion of cells to ECM proteins is important for regenerative processes and tissue homeostasis (Diller and Tabor, 2022).

Adhesion molecules, which play a fundamental role in this event, are proteins located on the cell surface that are involved in binding with other cells or the ECM, allowing the cells to arrange themselves to form tissues. The higher the cell affinity for the biomaterial, the more favorable its development (Fonticoli et al., 2023).

The protein expression levels of fibronectin, vimentin, E-cadherin and FAK, the principal markers of cell adhesion, were studied using immunofluorescence, western blotting, and gene expression analysis. Fibronectin is a high-molecular-weight glycoprotein of the ECM that binds to the membrane-spanning receptor proteins called integrins. Fibronectin is involved in cell adhesion, growth, migration, and differentiation. It is also important in processes such as wound healing and embryonic development (Pankov and Yamada, 2002).

Vimentin, a type III intermediate filament (IF) protein, is expressed in mesenchymal cells as a key cytoskeletal element. Vimentin plays a significant role in supporting and anchoring organelles to the cytosol. Moreover, it improves cell-cell interactions through its association with hemidesmosomes and desmosomes and increases integrin α5β1 binding to fibronectin (Marconi et al., 2021). E-cadherin is a calcium-dependent cell-cell adhesion molecule. In addition to suppressing cancer progression, it plays an important role in the growth, development, and intercellular adhesion of epithelial cells (van Roy and Berx, 2008). FAK is a closely related non-receptor protein and tyrosine kinase. It is involved in sending ECM-related signals relating to ECM, in association with cell anchoring and cytoskeletal actin. Integrin interactions with various extracellular matrix adhesive molecules are responsible for FAK activation (Salasznyk et al., 2007).

A significant increase in the protein expression of the principal markers involved in cell adhesion processes, such as fibronectin, vimentin and FAK, was detected in hPDLSCs cultured with BioR-Ps compared with hPDLSCs cultured with BioR-NPs, whereas E-cadherin was significantly expressed in hPDLSCs cultured with BioR-NPs compared with hPDLSCs cultured with BioR-Ps. Moreover, the results obtained by confocal microscopy were comparable with those obtained by western blotting.

VEGF family molecules are crucial in angiogenesis because of their ability to initiate the formation of vascular networks, cell proliferation and survival, gene expression, and nitric oxide production (Ferrara et al., 2003; Song et al., 2018).

However, many approaches have focused on the challenges of angiogenesis in tissue engineering (Zhang et al., 2021; Park et al., 2021). Constructing scaffolds capable of increasing the expression of VEGF and VEGF-R to promote angiogenesis and therefore regeneration remains a challenge in tissue engineering (Zhu et al., 2024). The increased expression of VEGF and VEGF-R indicates that the design of bioactive scaffolds with different geometries can be useful for angiogenesis induction and maintenance of tissue homeostasis (Diomede et al., 2020a; Zhu et al., 2024).

The results obtained by western blotting, confocal microscopy, and real-time PCR showed increased expression of VEGF and VEGF-R in cells cultured on BioR-Ps compared to that in cells seeded on BioR-NPs. These results demonstrate that holes in the membrane can influence the expression of angiogenic markers, such as VEGF and VEGF-R, providing better performance in terms of regenerative properties compared to non-held membranes. Furthermore, the results obtained from the ELISA assay, showing an increase in VEGF levels in supernatant hPDLSc + BioR-Ps compared to hPDLSCs + BioR-NPs, also confirm at a functional level the angiogenesis promoting effects of the BioR-Ps membranes compared to BioR-NPs.

Moreover, the morphological profile obtained by SEM investigation showed a marked interaction between the cells and the substrate through extended cytoplasmic elongation, and filopodia were more evident on the BioR-P surface. A variety of cells colonized the perimeter of the pores, showing a high affinity for the engineered biomaterial.

Despite the limitations of this *in vitro* study, relevant positive outcomes were obtained. Thus, these findings highlight the important role of holes on the membrane surface in promoting tissue regeneration in terms of cell growth, cell adhesion capacity, and the expression of molecules promoting angiogenesis progression. BioR-Ps, geometrically modified biomaterials, can be effective in several applications to ensure faster tissue regeneration. The presence of holes enables cell infiltration and neotissue deposition and promotes the selective release of biomolecules essential for clinical regeneration.

## Data Availability

The raw data supporting the conclusions of this article will be made available by the authors, without undue reservation.

## Supplementary material

SUPPLEMENTARY FIGURE S1 Human PDLSCs characterization. **(A)** Cytofluorimetric analysis of hPDLSCs, in the table, were reported the percentage of expression of each marker. Hematopoietic cell markers were not expressed. **(B)** Human PDLSCs observed under inverted light microscopy. **(C)** Oil red O-positive staining for lipid droplets were evident at cytoplasmic level after 28 days of differentiation time. **(D)** hPDLSCs showed a positive Alizarin Red S staining after 21 days of osteogenic induction. Scale bar: 10 μm.

SUPPLEMENTARY FIGURE S2 ELISA VEGF Level. **(A)** Hu VEGF standard curve. **(B)** ELISA VEGF level in hPDLSCs + BioR-NPs and hPDLSCs + BioR-Ps surnatants.
