# Supplementary figures and images for: Geometrically modified bovine pericardium membrane promotes the expression of molecules targeted for a faster integration and vascularization process
Source: Front Bioeng Biotechnol. 2024 Nov 13;12:1455215. doi: 10.3389/fbioe.2024.1455215 (PMC11598356; doi:10.3389/fbioe.2024.1455215)

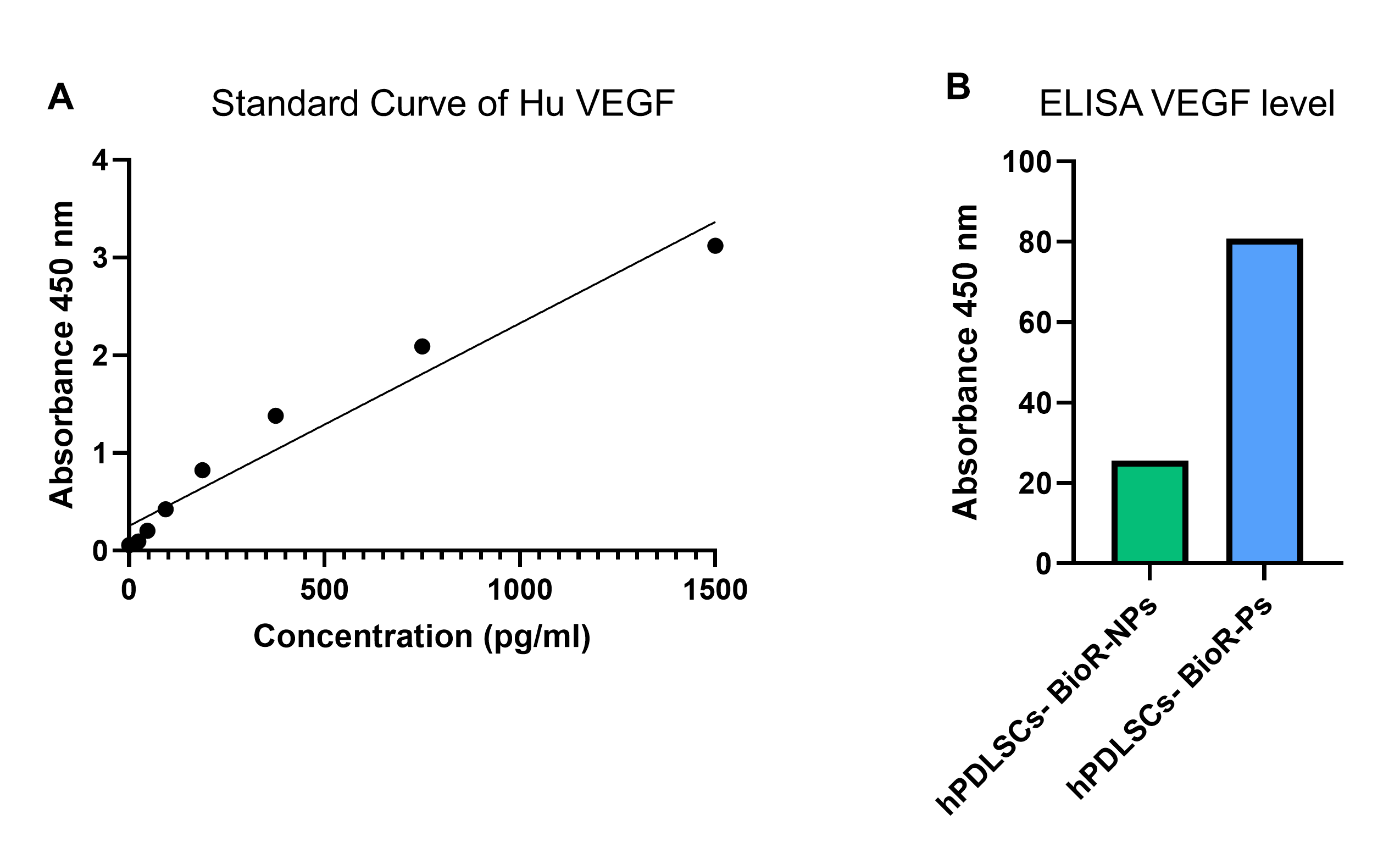

Supplement: Supplementary file 1 [file Image2.TIF]

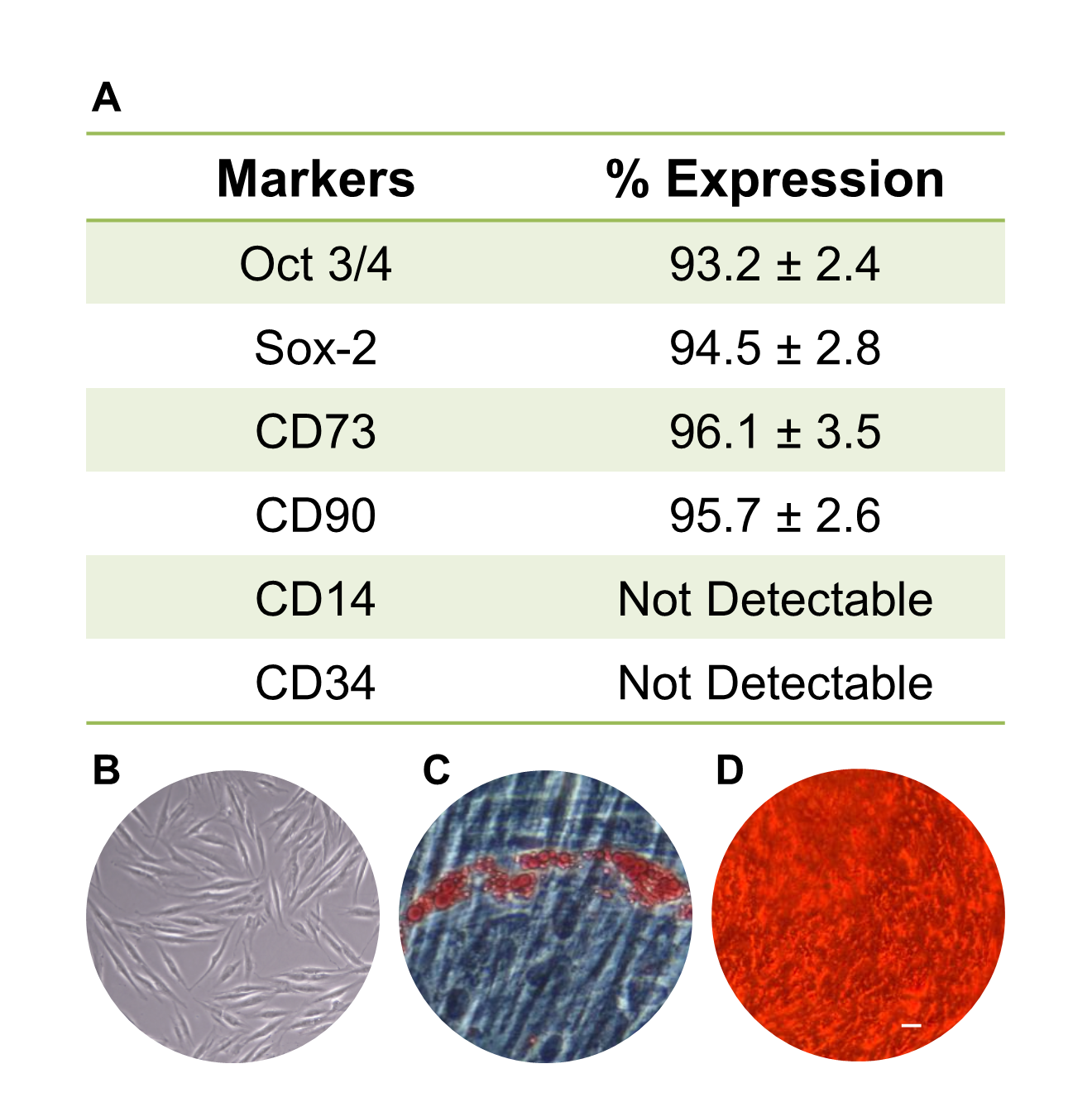

Supplement: Supplementary file 2 [file Image1.TIF]
